# Radiation-associated cardiovascular disease in patients with cancer: current insights from a cardio-oncologist

**DOI:** 10.1093/jrr/rrae068

**Published:** 2024-09-10

**Authors:** Masae Uehara, Norifumi Bekki, Taro Shiga

**Affiliations:** Department of Onco-Cardiology/Cardiovascular Medicine, The Cancer Institute Hospital of Japanese Foundation for Cancer Research, 3-8-31, Ariake, Koto-ku, Tokyo 135-8550, Japan; Department of Onco-Cardiology/Cardiovascular Medicine, The Cancer Institute Hospital of Japanese Foundation for Cancer Research, 3-8-31, Ariake, Koto-ku, Tokyo 135-8550, Japan; Department of Onco-Cardiology/Cardiovascular Medicine, The Cancer Institute Hospital of Japanese Foundation for Cancer Research, 3-8-31, Ariake, Koto-ku, Tokyo 135-8550, Japan

**Keywords:** radiation-associated cardiovascular disease, fibrosis, pericarditis, heart failure, valvular heart disease, ischemic heart disease

## Abstract

Radiation-associated cardiovascular disease (RACD), a complex disease characterized with pericarditis, myocardial damage, valvular heart diseases, heart failure, vasculopathy and ischemic heart disease, has a generally poor prognosis. While RACD may be acute, it often manifests in the late years or even decades following radiation exposure to the chest. With an increasing number of cancer survivors, RACD is likely to become an important issue in cardio-oncology. This review discusses pre-radiation therapy (RT) preparation, peri-RT patient management and long follow-up planning post-RT from a cardiology perspective. Additionally, a novel technique of stereotactic radiotherapy, which has been applied for the treatment of intractable cardiac arrhythmias, is presented. Appropriate patient examination and management during and after RT are essential to support patients undergoing cancer treatment to improve long life expectancy. A multidisciplinary team is needed to determine how to manage patients who receive RT to reduce RACD, to detect early phases of RACD and to provide the best treatment for RACD. Recent studies increasingly report advances in diagnosis using new equipment that has the potential to detect early phases of RACD, along with growing evidence for the optimal treatment for RACD. This review provides an overview of recent studies and guidelines to report on the latest findings, and to identify unresolved issues surrounding RACD that require validation in future studies.

## INTRODUCTION

Radiation therapy (RT) is an essential treatment for various types of cancer, including head and neck, breast, lung and prostate carcinomas, as well as lymphoma and skin cancers. It is performed to eradicate cancer cells, maintain cancer remission or reduce the risk of recurrence and, consequently, improve prognosis. Mediastinal lymphoma especially Hodgkin’s disease, breast cancer, esophageal cancer, and lung cancer are the target disease of mediastinal RT. Several previous reports have suggested the effectiveness of RT as an adjunct to chemotherapy and/or surgery for thoracic malignancy. RT after breast-conserving surgery for breast cancer reduces the 10-year risk of first recurrence by 15.7% and the 15-year risk of breast cancer death by 3.8% [1]. For Hodgkin’s lymphoma, the 5-year survival rate has been shown to improve to >85% in combination with RT and chemotherapy [2]. Recently, the combination of immune checkpoint inhibitors and RT has been shown to have the potential to increase the efficacy of therapy. However, there are crucial complications related to the heart and vasculature known as radiation-associated cardiovascular disease (RACD). Radiation exposure may influence all components of the heart and can provoke pericarditis, myocardial damage, valvular heart disease, ischemic heart disease, conduction system disorders and arrhythmias. Most RACD cases are observed after a long period after RT, and it correlates with poor prognosis and high mortality even if patients achieve cancer remission. Unfortunately, RACD remains underrecognized, and the exact rate and prognosis of RACD are not fully understood. In addition, most of the previous large observational studies on this issue were conducted using a previous generation of RT equipment with a wide area of radiation exposure, and the influence of RT using the new generation of RT equipment is unknown. As the number of cancer survivors increases with early detection due to advancements in diagnostic imaging and improved cancer treatments, further studies on the epidemiology, diagnosis and treatment of RACD are warranted. This review presents an overview of the current status of RACD by introducing recent articles and guidelines.

## Mechanism of RACD

RACD can be classified into acute onset (acute pericarditis and acute myocarditis) and chronic onset (chronic pericarditis, constrictive pericarditis, restrictive cardiomyopathy, ischemic heart disease, valvular heart disease, conduction system disorders and arrhythmias). Recently, acute RACD has become rare because of improved RT techniques with a more targeted application of dosage, which enables the reduction of radiation exposure to normal heart tissues. In contrast, the chronic onset of cardiovascular disease is still associated with high morbidity and mortality; thus, specific care is necessary in the long-term post-RT.

**Fig. 1:**
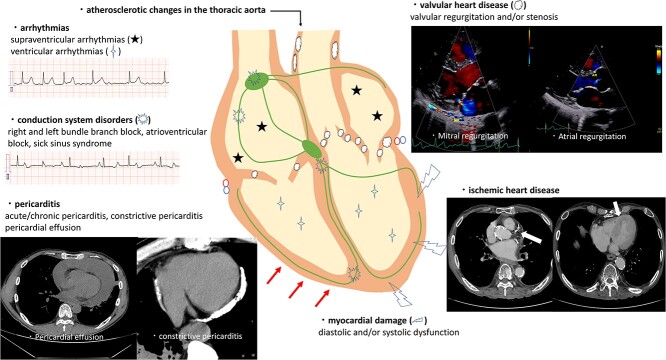
An image of radiation-associated cardiovascular disease.

The factors and mechanisms associated with RACD progression have not yet been fully elucidated. Currently, the main mechanism of RACD in the acute phase is considered to be the loss of endothelial cells and the subsequent inflammatory response that promotes vascular damage. The main mechanism of RACD in the chronic phase is through the progression of tissue fibrosis. The differentiation of fibroblasts into activated myofibroblasts occurs over a long period through inflammatory cytokines secreted by inflammatory cells. Activated myofibroblasts cause collagen accumulation and fibrosis, which leads to increased tissue stiffness. Loss of compliance in the myocardium results in reduced ventricular ejection fraction (EF) and impaired diastolic function, which contribute to the development of heart failure [3, 4]. Chronic inflammation in the pericardium causes thickening and calcifications in the pericardium with effusions, leading to loss of pericardial compliance and eventually manifesting as constrictive pericarditis [5]. Microvascular damage may impair the ability of the venous and lymphatic system of the heart to drain, resulting in chronic pericardial effusion. Damage to the conduction system can be caused by microvascular damage and fibrosis, accompanied by inflammation. In addition, direct damage to the conduction system cells by irradiation can cause disorders of conduction systems (Fig. 1) [6].

There is no established treatment against inhibiting the inflammatory response after RT, although statins may play a role in inhibiting tissue inflammation. A large number of studies have demonstrated the effectiveness of statins in reducing the risk of cardiovascular events in the general population. Guidelines for statin use for the primary and secondary prevention of cardiovascular diseases have been established based on accumulated evidence. Statins may also have a positive effect on patients post-RT because of their anti-inflammatory, antioxidant and anti-fibrotic effects, which may inhibit the progression of inflammation and fibrosis in heart structures after radiation exposure. However, there is a paucity of retrospective studies verifying the effectiveness of statin use in patients who underwent RT. A study from Walls *et al*. showed that statin therapy improved the overall survival in patients receiving RT for non-small cell lung cancer, especially those who had higher radiation doses and higher cardiac risks [7]. In contrast, Atkins *et al*. evaluated the effectiveness of statin use in 748 patients who underwent RT for non-small cell lung cancer and found that statin use was a significant independent predictor of all-cause mortality but not major adverse cardiac events (MACEs) [8]. Another study from Boulet *et al.* showed a significant 32% reduction in stroke and a strong trend toward reducing the composite outcome of cardiovascular and cerebrovascular events who were taking statin therapy in patients with cardiac issues and a history of RT [9]. Whether statin therapy is effective in patients who underwent RT and do not meet the criteria for statin use remains unclear. All of these studies were retrospective in nature; thus, large prospective randomized controlled studies are warranted to verify the effectiveness of statin use in these specific populations. Besides statin use, aspirin and colchicine may also play a role in preventing RACD due to their anti-inflammatory and anticoagulant properties. However, these drugs may be less beneficial than statins because of the high risk of bleeding in patients with cancers. There is still no evidence supporting those treatments [10].

## Risk of RACD

The risk of RACD comprises two components: radiation and patient factors. RACD is dose dependent, and a higher dose of RT increases the risk of RACD. However, a minimum radiation dose that is safe for the heart has not yet been established. Previous studies showed that high total cardiac radiation of >30 Gy or a daily dose fraction of >2 Gy/day leads to a high risk of RACD and that RACD is rare in patients who have received a cumulative dose of <4 Gy [3]. Recently, RT techniques have advanced dramatically. Changes from two-dimensional RT to three-dimensional conformal RT have been realized to provide effective radiation exposure to smaller fields. Intensity-modulated RT, volumetric-modulated arc therapy and stereotactic body RT can further reduce the dose delivered to adjacent normal tissues. Shielding and heart blocking are also effective techniques. Deep inspiration breath-holding and prone positioning are recommended in patients with left-sided breast cancer to reduce radiation exposure to the normal heart. In the future, proton therapy has the potential to reduce radiation exposure to the normal heart, although there are still limitations, such as higher costs and accessibility [11].

Generally, mean heart dose (MHD) is used as a surrogate parameter to evaluate the influence of radiation exposure on normal heart structures. There was a linear relationship between the estimated MHD and the risk of cardiovascular disease. However, the dose distribution to the heart varies significantly among patients undergoing modern RT. Some patients receive very high doses of irradiation to a small portion of the heart. Moreover, the normal heart is composed of various structures with different radiosensitivities. Considering these points, the radiation dose to the regional heart structures, rather than the whole-heart doses, seems to be more suitable for estimating the risk of future cardiovascular disease [12, 13]. Several studies have shown correlations between radiation doses to the regional heart structures, including the left anterior descending artery, left ventricular (LV) and cardiovascular diseases. Recently, a report from Lai *et al*. demonstrated that the relative volume of the left ventricle receiving 25 Gy with the cut-off point at 4% in patients with left-sided breast cancer was a more sensitive parameter to predict major coronary ischemic events as compared to MHD [14]. Another study from van den Bogaard *et al*. evaluated the cumulative incidence of acute coronary events during 7.6 years of the median follow-up in 910 females with breast cancer who received RT. They found that LV receiving 5 Gy was a better predictor for acute coronary events than MHD [15]. A large observational study in survivors of childhood cancer also demonstrated the utility of the mean radiation dose to the cardiac substructures rather than the mean whole-heart dose to identify the risk for late cardiac diseases [16].

Referring to patient factors, younger age at the time of RT, long-term follow-up after receiving RT and concomitant cardiovascular disease were risk factors for RACD. A history of receiving chemotherapy, notably anthracyclines and HER-2/neu receptor antagonist trastuzumab, has synergistic effects with radiation. A study from survivors of childhood cancer demonstrated the highest risk of cardiac disease in patients who had received both anthracycline and a heart radiation dose ≥30 Gy, where the risk was multiplied by 61.5 (95% confidence interval, 19.6–192.8) as compared to patients who have received neither of these treatments [17]. Another study from adult survivors of childhood cancer showed that heart radiation dose >1500 cGy and anthracycline exposure (≥250 mg/m^2^) had increased risk of valvular disease [18]. Cardiovascular risk factors including hypertension, diabetes mellitus, hyperlipidemia, smoking and kidney disease are also associated with an increased risk of RACD. In particular, these cardiovascular risk factors are associated with a high risk of vascular injury and heart failure but have a low impact on pericardial disease.

## Characteristics of RACD

It is difficult to diagnose RACD, as RACD mostly manifests long after RT. Since RACD is associated with high mortality and poor prognosis, early detection and therapeutic intervention are necessary. Primary RACD includes pericarditis, myocardial damage, valvular heart disease, ischemic heart disease, conduction system disorders and arrhythmias. All these disorders should be treated according to the cardiovascular guidelines in the general population. However, there are specific points to note regarding RACD. Physicians should consider the influence of radiation exposure on whole-heart structures and vasculature when considering treatments for cardiovascular diseases. The risk of operative treatment is higher in post-RT patients as fibrosis progresses in every normal irradiated tissue, with a high incidence of concomitant disease. Furthermore, patients who underwent RT may have worse outcomes after cardiovascular treatment than the general population. This section presents the characteristics, diagnoses and treatment of common RACD (Table 1).

**Table 1:** Time of onset, risks, major symptoms, and diagnosis methods of radiation-associated cardiovascular disease

	Time of onset				Risk stratification	Major symptoms	Diagnosis methods
	Just after RT	~Within one year after completion of RT	Later than one or more years after completion of RT (<five years)	More than five years after completion of RT			
**Associated medical personnel**							
Main attending physician	O/R/C	O/R/C	O/R/C and PCP	O/C and PCP			
MDT	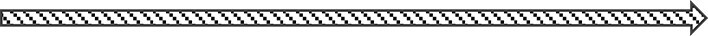						
**RACD**							
Acute pericarditis	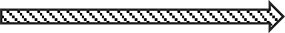				・ High radiation dose to the heart	chest pain, chest discomfort, shortness of breath, fever	ECG Chest X-ray TTE Cardiac CT Cardiac MRI
Acute myocarditis	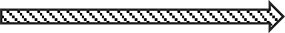				・ High radiation dose to the heart ・ Concurrent chemotherapy	chest pain, chest discomfort, shortness of breath, palpitation, syncope, fever	ECG Chest X-ray TTE Cardiac MRI Myocardial biopsy
Chronic pericarditis			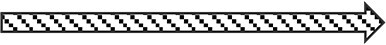		・ High radiation dose to the heart ・ History of receiving chemotherapy ・ Long-term after RT	chest discomfort, shortness of breath, edema	ECG Chest X-ray TTE Cardiac CT Cardiac MRI Right heart catheterization^a^
Disorder of conduction system	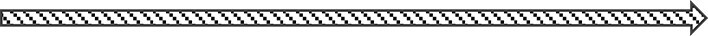				・ High radiation dose to the heart ・ Maybe existence of conduction disorders pre-RT ・ Cardiovascular risk factors^b^ ・ Long-term after RT	shortness of breath, dizziness, palpitation, syncope, edema	ECG Holter ECG Electrophysiological study
Arrhythmias	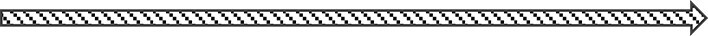				・ High radiation dose to the heart ・ Maybe existence of arrhythmias pre-RT ・ Cardiovascular risk factors ・ Long-term after RT	shortness of breath, dizziness, palpitation, syncope	ECG Holter ECG Electrophysiological study
Myocardial damage	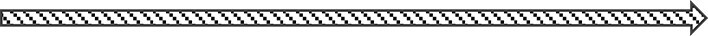				・ High radiation dose to the heart ・ Cardiovascular risk factors ・ History of receiving chemotherapy ・ Use of anthracyclines and/or trastuzumab ・ Myocardial dysfunction during the cancer treatment ・ Long-term after RT	shortness of breath, edema	ECG Chest X-ray TTE Cardiac MRI Cardiac CT^c^ SPECT Myocardial biopsy
Coronary artery disease				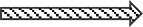	・ High radiation dose to the heart ・ Cardiovascular risk factors ・ Long-term after RT	chest pain, chest discomfort, shortness of breath	ECG TTE Cardiac MRI Cardiac CT SPECT PET Coronary angiography
Aortic disease				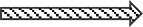	・ High radiation dose to the mediastinum ・ Cardiovascular risk factors ・ Long-term after RT	Asymptomatic	Chest CT Chest MRI
Valvular heart disease				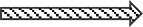	・ High radiation dose to the heart ・ Cardiovascular risk factors ・ Use of anthracyclines ・ Long-term after RT	shortness of breath, edema, palpitation, dizziness, syncope	Chest X-ray TTE TEE

RT: radiation therapy; O: oncologist; R: radiation oncologist; C: cardiologist; PCP: primary care physician; MDT: multidisciplinary team; RACD: Radiation-associated cardiovascular disease; ECG: electrocardiogram; TTE: transthoracic echocardiography; CT: computed tomography; MRI: magnetic resonance imaging; SPECT: single photon emission computed tomography; PET: positron emission tomography; TEE: transesophageal echocardiography.

^a^Whenever constrictive pericarditis is suspected.

^b^Cardiovascular risk factors including age, hypertension, hyperlipidemia, diabetes mellitus, smoking, and kidney disease.

^c^Cardiac CT can be used as an alternative to cardiac MRI when MRI is contraindicated.

### Pericarditis

Historically, pericarditis has been the most common complication of RT with an estimated rate of 20%. However, recently, the morbidity rate has been reduced to 2.5% with the advancement of RT techniques [19]. Radiation-associated pericardial disease is classified into acute pericarditis and chronic pericardial diseases, including delayed pericardial effusion and chronic pericarditis. Acute pericarditis is observed during or immediately after radiotherapy. Acute pericarditis is rarely observed due to the advantage of modern and advanced RT techniques. In contrast, chronic pericardial diseases are common [6, 20]. Acute pericarditis is strongly suspected when chest pain is observed, accompanied by electrocardiogram changes and pericardial effusion. It is treated with aspirin or nonsteroidal anti-inflammatory drugs and colchicine as the first-line treatment. Chronic pericarditis is defined as pericarditis lasting for >3 months and is usually less effective with anti-inflammatory drugs and colchicine [21]. Those patients may suffer from chronic pericardial effusion, and several cases may progress to constrictive pericarditis.

Chronic pericardial diseases can be diagnosed using electrocardiography (ECG), transthoracic echocardiography (TTE), cardiac computed tomography (CT) or cardiac magnetic resonance (CMR). A thickened pericardium and pericardial effusion can be observed using multimodal imaging. ECG-gated CT is preferable for diagnosing the amount of pericardial effusion, pericardial thickness and extent of pericardial calcification. CMR can identify the thickening of the pericardial layers and pericardial effusion through T1-weighted and cine imaging. Moreover, CMR can be used to evaluate inflammation and fibrosis in the pericardium using black-blood T2-weighted and contrast-enhanced T1-weighted imaging. CMR myocardial tagging and phase-contrast velocity imaging are recommended whenever constrictive pericarditis is suspected [22]. Patients with pericarditis due to RT may also have complicated myocarditis and myocardial fibrosis. In such cases, CMR is valuable for assessing the concomitant myocardial inflammation and fibrosis using cine imaging, black-blood T2-weighted imaging, as well as early and late gadolinium enhancement (LGE). Furthermore, T1 and T2 mapping techniques have the potential to detect diffuse myocardial inflammation and fibrosis with higher diagnostic accuracy.

Pericardial effusion can progress over a long period and is often asymptomatic. The cumulative incidence rates of pericardial effusion after chemoradiation therapy for non-small cell lung have been reported to be 31.4% at 1 year and 45.4% at 2 years. Pericardial effusion is strongly associated with higher radiation exposure to the heart [23]. The aggravation of pericardial effusion may subsequently manifest as cardiac tamponade, although this is observed only in limited cases. Cardiac tamponade can be diagnosed based on three signs: neck vein distension with elevated jugular venous pressure, pulsus paradoxus and diminished heart sounds on cardiac auscultation with a large amount of pericardial effusion. Pericardiocentesis should be considered in patients with a large pericardial effusion accompanied by hemodynamic compromise. A pericardial window is recommended in cases of recurrence.

The thickening of the pericardium with calcification can reduce its elasticity and exacerbate constrictive pericarditis, which is an intractable disease. Right heart catheterization is performed to confirm the diagnosis whenever constrictive pericarditis is suspected. Surgical treatment (i.e. pericardiectomy) may be considered for patients with persistent symptoms, even after titration of oral treatment, because there is no effective medical treatment for constrictive pericarditis. However, the mortality rate of post-pericardiectomy in radiation-induced constrictive pericarditis is high, and refractory cases are common. One study reported the prognosis of 98 patients who underwent pericardiectomy for constrictive disease. Five-year survivals were significantly lower in patients with post-RT as compared to postoperative and idiopathic constrictive disease (40.3%, 55.9% and 79.8%; *P* = 0.004) [24]. Considering a dismal outcome in patients with constrictive disease post-RT, it is crucial to make further efforts to reduce radiation exposure to the heart.

### Myocardial damage

RT to the mediastinum may cause diffuse fibrosis in the myocardium, leading to impaired ventricular contraction and dilation during the chronic phase. LV diastolic dysfunction is more frequently observed and often presents with heart failure with preserved EF than with heart failure with reduced EF. A previous study showed that 14% of patients with Hodgkin’s disease who underwent mediastinal irradiation developed diastolic dysfunction, which is associated with a worse prognosis [25]. Due to its anterior location, the right ventricular is also expected to receive the influence of radiation exposure. However, the details of RT-induced damage to the right ventricular have not been fully analyzed. Atrial dysfunction can also be observed along with atrial fibrosis, which may lead to atrial arrhythmias.

Anthracyclines are commonly used to treat breast cancer, although they can increase the risk of RT-related myocardial damage [17, 18]. These drugs damage the heart through a variety of pathophysiological mechanisms, including direct damage to the cardiomyocytes. The combined effects of radiation and anthracyclines may synergistically affect the myocardium. Regarding trastuzumab, which is commonly used as an anti-HER2 therapy for breast cancer, some reports have demonstrated its radiosensitizing effect on breast cancer cells. Whether it causes radiosensitization of normal cells remains unclear, although a study from Yi *et al.* has demonstrated that trastuzumab combined with irradiation causes more cardiotoxicity than irradiation or trastuzumab alone from *in vitro* and *in vivo* experiments [26]. Another study by Cao *et al*. showed a higher prevalence of LV diastolic dysfunction in patients who received trastuzumab combined with irradiation therapy as compared to RT alone (35.2% vs 19.7%, *P* = 0.061) [27]. Other reports have also indicated that patients with breast cancer who have received combined treatment of trastuzumab and RT are at a higher risk of diastolic dysfunction. Therefore, cardiovascular toxicity must be carefully considered in patients receiving concurrent trastuzumab or anthracycline, and adjuvant radiotherapy. During the long follow-up period, myocardial perfusion defects due to coronary artery and microvascular damage caused by RT may also lead to myocardial dysfunction. Furthermore, coexisting valvular heart disease may worsen LV systolic and diastolic functions.

TTE is the most useful and accessible examination method for detecting myocardial dysfunction. Three-dimensional (3D) echocardiography is preferred for evaluating cardiotoxicity in patients with cancers as it can more accurately evaluate LVEF and cardiac volumes than two-dimensional (2D) echocardiography. Recently, global longitudinal strain using speckle tracking has proven to be a promising technique for detecting subtle cardiac function changes, even in patients post-RT [28, 29]. Early detection of myocardial deformation may enable the initiation of treatment in the early phase, consequently preventing the progression of cardiac dysfunction.

**Fig. 2:**
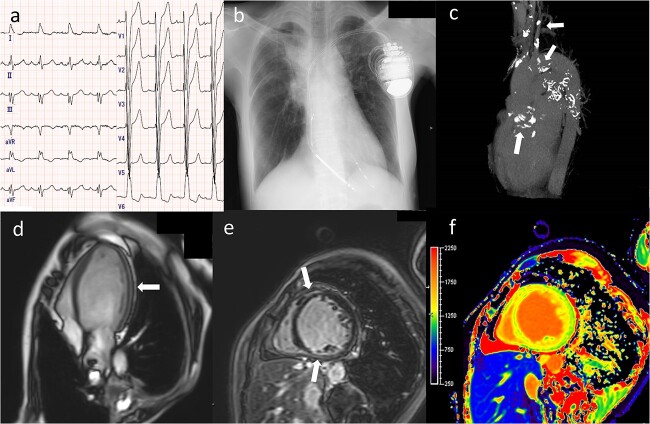
A 57-year-old woman with history of chemotherapy using anthracycline and radiation therapy for Hodgkin’s lymphoma admitted for heart failure. (a) An electrocardiogram showing a complete left bundle branch block; (b) a plain chest radiograph following CRTD implantation; (c) a CT scan before CRTD implantation showing calcification in the aorta, carotid arteries and aortic valve; (d) CMR before CRTD implantation showing enlargement of the LV with a reduced ejection fraction to 15.6%; (e) late gadolinium enhancement in the LV anterior and inferior wall; and (f) high native T1 in the septum suggesting diffuse myocardial damage. Abbreviations: CRTD, cardiac resynchronization therapy defibrillator; CT, computed tomography; CMR, cardiac magnetic resonance; LV, left ventricle.

When myocardial dysfunction is suspected, as well as in cases with poor-quality echocardiography windows, CMR should be performed to evaluate myocardial tissue characteristics. Cine imaging by CMR has high reproducibility for evaluating cardiac volume and EF, although it is difficult to detect the early stages of myocardial deformation. Most studies have failed to show a decline in LVEF with a short follow-up period, whereas a few studies have shown reduced LVEF in patients post-RT [30]. CMR was conducted >20 years after RT for lymphoma with relatively high radiation exposure to the heart in those studies [31, 32]. Recently, strain analysis using CMR has been shown to be a useful tool in detecting cardiac dysfunction in the early phase of cardiac disease. Few studies have demonstrated the utility of global strain analysis using CMR in patients who have undergone RT, although there are several conflicting results. With the advantage of RT techniques that induce reduced radiation exposure to the heart, regional rather than global strain analysis may be preferable for detecting radiation-associated cardiac dysfunction. An increasing number of studies have evaluated the use of LGE and T1/T2 mapping to detect myocardial fibrosis and inflammation after RT. LGE tends to be observed in patients who received a wide range of radiation exposures to the thoracic area (i.e. lymphoma and esophageal cancer), those who received old RT techniques and those who underwent CMR for a long period after RT. Similar to strain analysis, regional rather than global changes in T1/T2 mapping may be preferable for detecting diffuse myocardial damage in post-RT patients (Fig. 2).

Cardiac CT can be used as an alternative to CMR to evaluate cardiac function and characterize myocardial tissue. Recently, strain analysis and extracellular volume fractions have been evaluated using a new generation of CT equipment, although it is still under investigation, and its accuracy is not well established. Cardiac CT has a high negative value for coronary artery disease (CAD). Therefore, cardiac CT is recommended whenever CAD should be excluded as a cause of ventricular dysfunction. Lastly, radionuclide imaging may also play an important role in detecting myocardial damage after RT [33, 34]. Several reports have shown early myocardial metabolic disorders and myocardial perfusion defects assessed by nuclear imaging after RT. Cardiac imaging should be conducted more proactively to identify myocardial damage and assess the need for cardioprotective medications.

### Valvular heart disease

Valvular heart disease is a long-term complication of RT. It is well described by TTE, and periodic examinations are preferred whenever valvular heart disease is detected. Transesophageal echocardiography is essential when severe valvular regurgitation or stenosis is suspected and therapeutic intervention is considered. Valves are thickened with fibrosis, with or without calcification, resulting in valvular stenosis and regurgitation during long-term follow-up. Left-sided valves such as the mitral and aortic valves are more affected, and regurgitation is more common. Another characteristic appearance is a progression of the thickened and calcified aortomitral curtain (AMC; fibrous thickening separating the non-coronary and left coronary cusps from the anterior leaflet of the mitral valve). In a study of 173 patients with RACD who underwent cardiothoracic surgery, there was no association between AMC thickness and radiation dose, although there was a direct positive correlation with the time from the last radiation dose. Moreover, AMC thickness was found to be an independent predictor of long-term mortality in patients with RACD who underwent cardiothoracic surgery [35].

Patients with post-RT develop valvular heart disease at a younger age than the general population. It is also characterized by a higher rate of complications with other valvular diseases and concomitant cardiovascular diseases, which may be associated with a worse prognosis. Several previous studies have demonstrated that the short- and long-term outcomes of open-heart surgery in patients with radiation-related heart disease are worse than those in the general population. Wu *et al*. evaluated long-term survival in 173 patients with radiation-associated heart disease (RAHD) and 305 matched controls who underwent cardiothoracic surgery. They showed that the presence of RAHD, increasing EuroSCORE, and lack of β-blockers were associated with increased mortality [36]. Similarly, patients with post-RT and who underwent surgical aortic valve replacement for severe aortic stenosis (*n* = 172) showed increased longer-term mortality as compared to a matched cohort (*n* = 172) [37]. Respiratory complications and the presence of myocardial damage due to RT were also shown to influence worse outcomes in these specific populations. Moreover, post-RT patients are at high risk for cardiac surgery because of inflammation and fibrosis in the mediastinal tissues. Bleeding increased during the operation, and healing after the operation was intractable. Recently, transcatheter aortic valve replacement (TAVR) for severe aortic valve stenosis and transcatheter valve repair for severe mitral and tricuspid regurgitation have been established. In general, TAVR is recommended for patients with higher age and high or intermediate operative risk. Recently, a prospective randomized study reported that TAVR procedures for patients with lower surgical risk also had no inferiority compared with traditional surgical aortic valve replacement [38]. There is growing evidence of the safety, effectiveness and durability of TAVR, and thus, minimally invasive heart procedures may reduce poor outcomes in patients with post-RT.

In patients with a history of RT, Zhang *et al*. showed lower mortality, less postoperative atrial fibrillation (AF) and shorter duration of hospital stay in patients who underwent TAVR than those who underwent surgical aortic valve replacement (SAVR) [39]. Similarly, a large observational study compared the outcomes of TAVR (*n* = 2170) and SAVR (*n* = 1505) who had a history of mediastinal radiation exposure and showed lower in-hospital mortality in patients who underwent TAVR [40]. Another study showed no differences in the early and late outcomes in post-TAVR between patients with a history of RT (*n* = 26) and matched controls (*n* = 26) [41]. In contrast, the effectiveness of transcatheter edge-to-edge repair (TEER) for severe mitral regurgitation (MR) in post-RT patients is controversial. TEER is only considered in patients with symptomatic severe MR, those who are disqualified from surgical treatment and those who fulfill the echocardiographic criteria with an appropriate valve morphology [42]. MR due to RACD complicates calcification in the mitral annulus, sub-valvular apparatus and aortomitral curtain and occasionally develops other valvular diseases. Moreover, patients with RACD have a risk of developing mitral stenosis long after treatment with MR. In this regard, TEER for post-RT patients may be less effective than that for the general population. One study showed a worse prognosis in patients with a history of cancer (*n* = 82), including 21 patients with post-RT, compared to non-cancer patients (*n* = 364) who underwent TEER for severe MR. Estimated 1-year mortality was 20.2% in patients with a history of cancer versus 9.2% in non-cancer patients [43]. There is a paucity of data regarding TEER in patients with post-RT. Therefore, additional studies are warranted to determine the optimal treatment regimen for these patients.

### Vascular injury

RACD is observed in various types of vascular diseases, including microvascular, coronary artery and macrovascular diseases, which develop 10–30 years after radiation treatment. RT-associated CAD is the most common disease, and its mechanism is considered to be as follows: vascular endothelial cells are damaged by RT, leading to a proinflammatory state. Blood vessels are damaged by oxidative stress, generation of reactive oxygen species and cytokine release, which disrupt DNA strand integrity. Subsequently, fibrin deposition and platelet aggregation occur, which accelerates atherosclerosis [4, 5]. The lesions are more often observed in the ostia of the coronary arteries in the left main trunk, left anterior descending artery and right coronary artery. Stenotic lesions tend to be diffuse, long and appear to be concentric. One study showed the morphological plaque characteristics using optical coherence tomography in patients with acute coronary syndrome among patients with cancer (*n* = 63) as compared to non-cancer patients (*n* = 373). This study includes four patients who received RT and showed that plaque erosion and calcified nodules rather than plaque rupture were more often observed in patients with cancer. The plaque characteristics and mechanisms of acute coronary syndrome might be different in patients with cancer and those who received RT [44]. Further studies are necessary to identify high-risk patients with high-risk plaques in patients with a history of cancer treatment.

The incidence of CAD depends on the presence of cardiovascular risk factors, atherosclerosis, age and duration of RT [45]. Salz *et al*. suggested that mediastinal RT increases the risk of myocardial infarction in non-Hodgkin’s lymphoma survivors, especially those with cardiovascular risk factors [46]. They demonstrated that the predicted absolute 10-year risk of myocardial infarction was 2.0% in patients without a history of mediastinal RT and no cardiovascular risk factors, 3.9% in patients after mediastinal RT with no cardiovascular risk factors and 10.2% in patients after mediastinal RT with cardiovascular risk factors. Additionally, the radiation dose, location and range of radiation may influence the development of CAD. A study evaluating 2168 women who underwent radiotherapy for breast cancer showed that the risk of major coronary events increased linearly with the mean dose to the heart [47]. A large observational study from the Childhood Cancer Survivor Study showed that historical reductions in radiation exposure to the heart reduced the risk of CAD among adult survivors of childhood cancer [48].

The examination and treatment of CAD in patients with a history of RT follow the conventional guidelines. Immediate angiography is recommended whenever ST-elevation myocardial infarction or non-ST-elevation acute coronary syndrome with high-risk features is suspected. Resting ECG, biochemistry, chest radiography and TTE were preferred to assess baseline conditions. Regarding angina and suspected CAD, stress ECG, stress echocardiography, coronary CT angiography, stress CMR, positron emission tomography or single-photon emission computed tomography are selected according to the pre-test probability and clinical likelihood of CAD [49]. If coronary stenosis is strongly expected from those examinations, coronary artery angiography and fractional flow reserve should be performed to consider the treatment. Treatment of CAD is divided into three types, that is, oral treatment alone, percutaneous coronary intervention (PCI) and coronary artery bypass grafting. When there is evidence of myocardial ischemia with coronary artery stenosis, PCI or coronary artery bypass grafting (CABG) is considered according to the severity of the CAD. Reed *et al*. evaluated long-term mortality post-PCI in patients with radiation-associated CAD (*n* = 157) when compared with age, sex, type of PCI and target artery matched controls (*n* = 157) with an average follow-up duration of 6.6 ± 5.5 years after PCI. They demonstrated that the history of RT was an independent factor for predicting all-cause and cardiovascular mortality [50]. In contrast, a study from Fender *et al*. showed conflicting results that there were no significant differences in long-term mortality between post-RT and controls after PCI [51]. Using bare metal stents is also an independent factor for predicting all-cause mortality, and thus drug-eluting stents should be considered [50]. Regarding the revascularization of the coronary artery in left main stenosis lesions or severe three-vessel diseases, PCI may be preferable as compared to CABG in patients with a history of RT because those specific patients have a high risk of open-heart surgery as mentioned in valvular heart disease.

The coronary artery calcium score evaluated using ECG-gated non-contrast CT is a proven tool for the cardiovascular risk assessment over traditional CAD risk factors. A higher coronary calcium score suggests a higher risk of MACE in the future [52]. Coronary artery calcium scoring of non-gated CT has been shown to correlate well with ECG-gated CT and has been proven to be an alternative method to evaluate coronary artery calcium for risk stratification. Several studies have demonstrated the effectiveness of the coronary calcium score evaluated by RT planning CT scans in patients with breast cancer [53]. A study from Roos *et al*. showed a rise in acute coronary events in patients with breast cancer and higher coronary artery calcium levels, as evaluated by pretreatment CT scans, even after correction for confounding factors. The median follow-up was relatively long and it was 7.5 years in this study [54]. A larger study from Gal *et al*., which evaluated 15 915 patients with breast cancer receiving RT, demonstrated an increased risk of cardiovascular disease in patients with increasing coronary artery calcium score after a median follow-up time of 51.2 months [55]. Contrary, coronary artery calcium scores did not have a statistically significant impact on overall survival in patients with non-small cell lung cancer with a median follow-up time of 7 years [56]. The conflicting results were probably due to poorer 5-year survival in patients with non-small cell lung cancer as compared to those with breast cancer. A recent study by Tjessem *et al*. demonstrated a similar coronary artery calcium score distribution between post-RT patients with breast cancer and an age-matched general population. There was no excess coronary artery calcium in breast cancer survivors treated with RT, and the contribution of radiation dose to coronary artery calcium could not be demonstrated in this study [57]. It is still not clear whether RT accelerates coronary artery calcium burden over the presence of other underlying cardiovascular disease risk factors for a long period [58, 59]. Additionally, it is unknown whether preventive therapies, including statin and/or aspirin use, are beneficial in patients with moderate or severe coronary artery calcium scores. Further research and discussion are required to determine which post-RT patients with cancer are at high risk of CAD in the future and how to treat these patients to prevent cardiovascular disease (Table 2).

**Table 2:** Studies evaluating coronary calcium score for risk stratification in patients who received radiation therapy

Study	Type of study	Population	CT imaging for evaluating CAC	Median follow-up	Techniques of RT	Cancer treatment	Results
Kim *et al*. 2022 [50]	Retrospective study	Breast cancer who received mastectomy with (*n* = 511) and without (*n* = 600) adjuvant RT	– RT group: non-ECG-gated CT scans for RT planning – No-RT group: PET-CT scans without ECG synchronization	9.3 y	− 50.4 Gy dose at 1.8 Gy per fraction to the whole breast or chest wall − 35.9% received tumor bed boost – MHD (median): 3.4 Gy	– Anthracycline use: RT group: 59.5%, no-RT group: 51.5% – Anti-HER2 treatment: RT group: 13.9%, no-RT group: 9.2%	In a multivariate Cox regression analysis, CAC score > 0 and MHD >3 Gy was a risk factor for acute coronary events
Roos *et al*. 2017 [51]	Retrospective study	939 patients with breast cancer (stages I–III) or DCIS	– Non-ECG-gated CT scans for RT treatment	7.5 y	− 50.4 Gy dose in 28 fractions to the whole breast with a boost dose of 14 or 16.8 Gy in 28 fractions – MHD (median): 2.36 Gy	– Breast-conserving surgery followed by RT – Systemic treatment Chemotherapy only: 11.0%, Endocrine therapy only: 17.0%, Both: 46.5%	The cumulative incidence of acute coronary events was higher in patients with CAC score >100 compared with CAC zero even after correction for confounding
Gal *et al*. 2021 [52]	Retrospective study	15 915 patients who received RT for primary breast cancer (pathologic tumor stages: DCIS/T0 13.5%, T1 59.9%, T2 21.6%, ≥T3 3.6%)	– Non-ECG-gated CT scans for RT planning	51.2 mo	– Locoregional with boost: 4.3%, without boost: 7.2% – Local with boost: 28.3%, without boost: 26.3%	– No chemotherapy: 60.8% – Anthracyclines use: 27.2% – No hormonal therapy: 55.1%	Cardiovascular disease risks increased from 5.2% in patients with no CAC to 28.2% in patients with CAC scores >400 during follow-up
Olloni *et al*. 2023 [53]	Retrospective study	644 patients with non-small cell lung cancer (stages ≤IIB: 12%, ≥IIIA and recurrence: 82%)	– Non-ECG-gated non-contrast-enhanced RT planning CT	7 y	– 2- to 2.75-Gy fractions with five weekly fractions – Mostly, 60–66 Gy in 30–33 fractions – RT technique (3D-CRT: 9%, IMRT: 41%, VMAT: 50%)	– Not stated	No association between elevated CAC and overall survival
Polomski *et al*. 2023 [55]	Cross-sectional matched cohort study	− 97 patients with HL, referred for CCTA >10 years after RT (stages I–II: 74.2%, III–IV: 25.8%) – 97 matched non-cancer control	– ECG-triggered CT using 320-row volumetric scanner for evaluating asymptomatic CAD	8.5 y after CCTA	– Mantle field irradiation: 35.1% – Mediastinal RT: 47.4% – Subtotal nodal RT: 13.4% – ≥ 35 Gy: 74 patients, <35 Gy: 21 patients	− 83.5% of the patients were treated with chemotherapy – Anthracycline use: 70.1%	CAC score >0 and non-obstructive coronary artery stenosis were significantly higher with more cardiovascular events in HL survivors
Atkins *et al*. 2022 [56]	Retrospective study	428 patients with non-small-cell lung cancer (stages II–III)	– Non-ECG-gated, non-contrast-enhanced RT planning CT – CAC was quantified by a deep learning model	18.1 mo	– 3-dimensional conformal RT or IMRT – MHD (median): 11.0 in CAC = 0, 12.2 in CAC ≥1	– Radiotherapy plus chemotherapy and/or surgery	Elevated CAC was associated with all-cause mortality, although MACE was not in the setting of limited events

CT: computed tomography; CAC: coronary artery calcium; RT: radiation therapy; ECG: electrocardiogram; PET-CT: positron emission tomography–computed tomography; y: years; MHD: mean heart dose; DCIS: ductal carcinoma *in situ*; mo: months; 3D-CRT: three-dimensional conformal radiation therapy; IMRT: intensity-modulated radiation therapy; VMAT: volumetric-modulated arc therapy; HL: Hodgkin lymphoma; CCTA: coronary computed tomography angiography; CAD: coronary artery disease; MACE: major adverse cardiac event.

Macrovascular diseases can be observed in the thoracic aorta and aortic arch, and often manifest as atherosclerotic changes. Mediastinal radiation is recognized as a cause of porcelain aortas and may be associated with increased mortality. Localized thrombi, which may lead to vascular occlusion or embolic stroke, are observed in some cases.

### Disorder of conduction system and arrhythmias

The causes of disorders of conduction system after RT can be classified into primary and secondary causes. Radiation exposure to the heart induces fibrosis in the atria, conduction pathways and myocardium, resulting in the development of an atrioventricular block, sick sinus syndrome, AF, premature atrial and ventricular contractions and ventricular tachycardia long after RT. In particular, the right bundle branch block is more often observed when the right bundle is located anteriorly to the heart. Additionally, disorders of the conduction system and arrhythmias can develop secondary to other RACD, including ischemic heart disease, cardiomyopathy and valvular heart disease.

AF is the most common supraventricular arrhythmia that can be exacerbated by atrial inflammation and fibrosis. RT for esophageal cancer is expected to be a risk factor for AF as the atrium is located adjacent to the esophagus. Several studies have demonstrated an increased risk of AF in patients with esophageal cancer who underwent neoadjuvant chemoradiation therapy followed by surgical resection. A study by Miller *et al*. revealed that nearly 20% of patients with esophageal cancer developed AF after RT initiation and that the incidence of AF was associated with worse outcomes [60]. AF was significantly increased in patients with higher radiation doses, although the safety radiation dose to reduce the risk of AF is unknown [61]. Additionally, a report from Haq *et al*. showed that a history of mediastinal RT is an independent risk factor for AF recurrence after an ablation procedure in patients with breast cancer [62]. Inflammation of the surrounding tissues due to the surgery, and direct influence of RT to the heart may induce inflammation and fibrosis in the atrium, which provides the substrate of AF. Autonomic dysfunction due to radiation exposure of the autonomic nerves and the side effects of chemotherapy may also be associated with the incidence of AF, although the exact pathogenesis of AF post-RT has not been fully elucidated.

The etiology of bradyarrhythmia in post-RT patients is thought to be a direct radiation injury and subsequent fibrosis of the conduction system. There have been several reports of RACD presenting with bradyarrhythmia requiring pacemaker therapy. However, a recent large study from Denmark revealed no increased risk of cardiovascular implantable electronic devices (CIEDs), implying the presence of severe conduction disorders during a median follow-up time of 11.7 years, in patients with early-stage breast cancer who underwent RT. This study was conducted in patients with breast cancer who underwent less radiation exposure to the heart as compared to those with Hodgkin’s lymphoma and esophageal cancer [63]. These results may be influenced by the patient’s background, including the type of cancer, cumulative dose of radiation exposure to the heart, comorbidities and patient age. Additional studies are warranted to stratify the risk of developing conduction disorders after RT and plan the optimal timing for follow-up examinations.

Autonomic dysfunction is another factor associated with the development of arrhythmias in patients after mediastinal RT, particularly chest and neck radiation. High sinus and abnormal heart rates following exercise due to autonomic dysfunction in patients after RT have been demonstrated by Groarke *et al*. The mechanism is considered to be the destruction of the superficial vagal ganglia and pulmonary vascular remodeling by radiation exposure, although detailed studies have not been conducted, and the mechanism is unknown [64].

## Management of CIEDs during RT

The number of patients who receive CIEDs for heart disease is increasing because of the aging population and advances in the treatment of heart disease. CIEDs include a cardiac pacemaker for bradyarrhythmia, an implanted cardioverter-defibrillator (ICD) for life-threatening ventricular arrhythmia and cardiac resynchronization therapy (CRT-P or CRT-D) for severely reduced cardiac function. RT in patients with CIEDs is associated with a risk of device malfunction. Programming resets, signal interference causing alterations in the sensing and pacing thresholds, inappropriate ICD shock and permanent damage to the device are observed. A retrospective study that evaluated the incidence of CIED malfunction during 249 courses of RT (123 pacemakers and 92 ICDs) showed that 7% occurred device malfunction [65]. Another study from Denmark reported a 3.1% rate of CIED malfunction among 453 RT courses [66]. Risk stratification of device malfunctions before RT is recommended so to plan the appropriate device management and device monitoring during RT. The risk of device malfunction is expected to increase with an increase in radiation exposure. The cumulative dose to the device is recommended not to exceed 2 Gy to a pacemaker and 1 Gy to an ICD as per the 2022 European Society of Cardiology (ESC) guidelines on cardio-oncology, although a safe radiation dose has not yet been determined [67]. Moreover, neutron-producing RT, which occurs with increasing energy photons >10 megavolts (MV), electron energy of >20 MeV and proton therapy, causes damage to the devices [68]. Several studies suggest that neutron-producing RT is more associated with device malfunction as compared to direct incident radiation dose to the CIEDs; thus, the use of non-neutron-producing RT is preferable in patients with CIEDs. In addition to the area of radiation exposure and radiation energies, a maximum cumulative incident dose >5 Gy, pacing dependence and frequent ICD therapies are risk factors for device malfunction in patients receiving thoracic RT.

Despite risk stratification, all patients with CIEDs are recommended to undergo device evaluation before and after RT. Cardiologists and electrophysiologists in a multidisciplinary team should consider whether reprogramming of pacemakers and ICDs is preferable during RT and whether the patient needs to check the CIEDs weekly during RT sessions according to the risk assessment. ECG and/or pulse oximetry monitoring and external pacing during RT sessions are recommended for patients at a high risk of device malfunction. In the case of tumors located adjacent to the CIEDs and when they interfere with the management of RT, device relocation may be discussed with the multidisciplinary team, although it is not recommended when the cumulative radiation dose is <5 Gy. Evaluation of CIED may be considered for high-risk patients 1 to 6 months after RT. However, late CIED malfunctions are reported to be low; thus, it may be unnecessary to follow up CIEDs for long periods after RT in addition to regular medical care.

## Long follow-up after RT

RACD can be observed later in life and threatens the prognosis of cancer survivors. Significant CAD can be observed for >5 to 10 years, and hemodynamically significant valvular heart disease is common for >10 years after RT [69]. RACD risk increases with the length of time since receiving RT; hence, a long follow-up for detecting RACD is crucial, especially for the cancer survival of children and adolescents who underwent RT in combination with chemotherapy. However, no prospective studies have evaluated the optimal timing of cardiovascular examinations according to the cardiovascular risk, and evidence is lacking.

Based on the ESC guideline, long-term follow-up surveillance for cancer survivors is recommended to be conducted according to the cardiovascular risk assessment including the radiation dose to the heart, total cumulative dose of doxorubicin, baseline cardiovascular toxicity risk, development of cancer therapy–related cardiovascular dysfunction during the cancer treatment, cardiovascular risk factors and a history of receiving hematopoietic stem cell transplantation [67]. The annual history of cardiovascular disease should be checked and cardiovascular risk should be assessed with examinations including biomarkers and electrocardiogram. The presence of risk factors for cardiovascular disease synergistically contributes to RT and increases the risk of coronary events. Therefore, cardiovascular disease risk factors, including hypertension, diabetes mellitus, lipid abnormalities and smoking, should be aggressively optimized. The measurement of cardiac biomarkers, such as brain natriuretic peptide and troponin, is also considered to be useful for detecting cardiac events in the early phase. Cardiovascular imaging is required whenever there are signs or symptoms suggestive of cardiovascular disease.

In asymptomatic patients, screening for CAD is recommended to be initiated <5–10 years after RT using a functional noninvasive stress test and reassessed every 5 years thereafter [69]. Coronary calcium scoring and coronary CT may also have the potential to be used for screening CAD after RT. Screening for valvular heart disease by TTE is recommended to initiate 5 years after RT with reassessment every 5 years thereafter [70]. Whenever abnormal findings are detected, frequent follow-up is necessary with consideration of transesophageal echocardiography in patients who progress to severe valvular disease. TTE evaluation of cardiac function in asymptomatic patients without cardiac dysfunction during RT is also recommended to start 5 years after RT. In ESC guidelines, patients who received RT >25 Gy MHD or RT >15–25 Gy MHD in combination with doxorubicin ≥100 mg/m^2^ are classified as very high-risk patients who are recommended to undergo TTE at years 1, 3 and 5 after completion of cardiotoxic cancer therapy and every 5 years thereafter [67]. Screening for pericardial disease aligns with TTE for evaluating valvular heart disease and cardiac function. However, TTE is insufficient for evaluating the overall pericardium. Thus, additional examinations, including CMR and cardiac CT, are preferable when pericardial disease is expected.

Education for cancer survivors is the most important factor in continuing examinations for screening for cardiovascular diseases associated with radiation exposure. For cancer survival in children and adolescents, the establishment of a system for the smooth transfer of management from the pediatric department to the adult department to ensure continuous follow-up is also important.

## Cardiac radiation for treatment of arrhythmia

Stereotactic radiotherapy can deliver a high dose of radiation to the target area with minimal exposure to surrounding normal tissues. Recently, stereotactic radiotherapy techniques have been applied for the treatment of cardiac arrhythmias, including ventricular tachycardia (VT) and AF [71]. Catheter ablation has also been established as an effective and safe procedure for patients with atrial and ventricular arrhythmias. However, the procedure has limitations in patients whose arrhythmia substrate is located in inaccessible areas, for example, mid-myocardial and epicardial surfaces, and whose anatomy is difficult. Recent studies have shown the effectiveness and safety of stereotactic ablative radiotherapy in patients with drug-refractory VT [72, 73]. The reduction in VT episodes has been demonstrated with a low incidence of serious adverse events, although there are several reports showing conflict results [74]. In the future, this technique has the potential to be an effective procedure for refractory arrhythmias. However, the influence of high-dose radiation exposure on focal areas is still unknown, and the late effects of RACD, as mentioned above, are also a concern. All previous trials and case reports in this field have been conducted with small sample sizes and short follow-up periods. Large clinical trials with control arms and longer follow-up periods are essential to verify the effectiveness and safety of the procedure for establishing treatment.

## CONCLUSION

Various clinical research has been presented related to RACD, although there is still a lack of evidence regarding the incident rate of RACD with advanced techniques of RT, risk factors of RACD, the optimal management of long follow-up and the appropriate treatment for RACD. Although RT is an essential cancer treatment, the number of patients undergoing RT in Japan remains low. However, this number is expected to increase in the future, owing to its superiority in cancer treatment. Healthcare professionals should thoroughly understand the issues surrounding RACD, collaborate across disciplines, prioritize patient education and anticipate long-term benefits for cancer survivors by accumulating evidence.

## FUNDING

The authors have no funding sources to declare.

## CONFLICT OF INTEREST

The authors have no competing interests to disclose.
